# An animal model of buccal mucosa cancer and cervical lymph node metastasis induced by U14 squamous cell carcinoma cells

**DOI:** 10.3892/etm.2013.938

**Published:** 2013-01-30

**Authors:** XIN ZHAO, LIANG PANG, YU QIAN, QIANG WANG, YONG LI, MINGYI WU, ZILAN OUYANG, ZHI GAO, LIHUA QIU

**Affiliations:** 1 Department of Biological and Chemical Engineering, Chongqing University of Education, Chongqing 400067;; 2 Department of Oral and Maxillofacial Surgery, The Affiliated Hospital of Stomatology, Chongqing Medical University, Chongqing 401147;; 3 Department of Stomatology, The Second Affiliated Hospital, Chongqing Medical University, Chongqing 400010, P.R. China

**Keywords:** buccal mucosa cancer, ICR mice, U14 squamous cell carcinoma cells, metastasis

## Abstract

The buccal mucosa is the site with the highest risk of contracting a malignancy in habitual betel quid chewers who expose the buccal mucosa to high doses of carcinogens. Of all oral cancers, those of the buccal mucosa are associated with the poorest prognoses. Therefore, it would be helpful to have an animal model to evaluate new treatment modalities for buccal mucosa cancer. In the present study, we evaluated whether the imprinting control region (ICR) mouse animal model could be employed as a cancer model for buccal mucosa cancer. Sixty male ICR mice were randomly divided into two groups, a normal group (n=10) and a cancer-induced group (n=50). Each mouse in the cancer group was inoculated with 0.05 ml U14 cancer cell suspension (1×10^7^/ml) on the buccal mucosa. Histological staining and gene expression assays revealed that neck lymph node metastasis animal models were established. After 20 days, the cheek tumor formation rate of the ICR mice reached 100%. Furthermore, the neck lymph node metastasis rate was 53%. We identified that U14 cells produce strong metastasis in ICR mice. Metastasis of the tumor to the lymph node began with carcinoma metastasis encroaching on the marginal sinus. Then it infiltrated to the cortex and medulla and the infiltration continued until the normal lymph node structure was completely damaged. This animal model may be employed in medical research on buccal mucosa cancer and cervical lymph node metastasis. In conclusion, our findings indicate that U14 cell-induced mouse buccal mucosa cancer may be a potential cancer model for human buccal mucosa squamous cell carcinoma.

## Introduction

Oral squamous cell carcinoma is a type of cancer that usually develops on the squamous or epithelial cells that cover the lips and oral cavity. The malignant or cancerous cells are usually located on the floor of the mouth or on the surface of the tongue. These cancerous cells also originate on the lower lips and palate or the tonsillar area of the oral cavity (1). It is considered that squamous cell carcinoma develops from the keratinizing or malpighian epithelial cells, as the presence of keratin has been observed in the malignant cells. It is one of the most prevalent types of oral and pharyngeal cancers (2). Squamous cells are the main component of the epidermis of the skin and this cancer is one of the major forms of skin cancer. Squamous cell carcinoma is the second-most common cancer of the skin (3). Squamous cell cancers of the skin in individuals on immunotherapy or suffering from lymphoproliferative disorders tend to be much more aggressive, regardless of their location (4).

Imprinting control region (ICR) mice are widely used in cancer studies. Animal models facilitate the development and testing of new approaches of research on disease prevention and treatment, identification of early diagnostic markers and novel therapeutic targets, as well as provide an understanding of the *in vivo* biology and genetics of tumor initiation, promotion, progression and metastasis (5). The U14 mouse tumor is a squamous cell carcinoma that was ectopically induced by treating the uterine cervix with 20-methylcholanthrene. The tumor was established and maintained by the Chinese Academy of Medical Sciences and Peking Union Medical College in 1958 (6). Following transplantation of U14 cells, the incidences of lymphatic and pulmonary metastasis were 95 and 80%, respectively, and the average survival time of mice with the tumor was 27 days. U14 cells are widely used in studies of tumor invasion, metastasis, recurrence and drug screening. Establishment of a cultured tumor cell line, which forms a tumor *in vivo*, would be helpful for the study of tumor biology on a cellular and molecular level (7–9).

In this study, we established an animal model by inducing mice with U14 squamous cell carcinoma cells, for *in vivo* evaluation of buccal mucosa cancer. The model successfully induces buccal mucosa cancer in mice and the application of the model may improve the experimental results of future research on buccal mucosa cancer.

## Materials and methods

### Animals

Seven-week-old male ICR mice (n=60) were purchased from the Experimental Animal Center of Chongqing Medical University (Chongqing, China). They were maintained in a temperature-controlled facility (temperature 23±1°C, relative humidity 50±5%) with a 12-h light/dark cycle. The mice had unlimited access to a standard mouse chow diet and water.

### Cancer cell preparation

**Figure 1:**
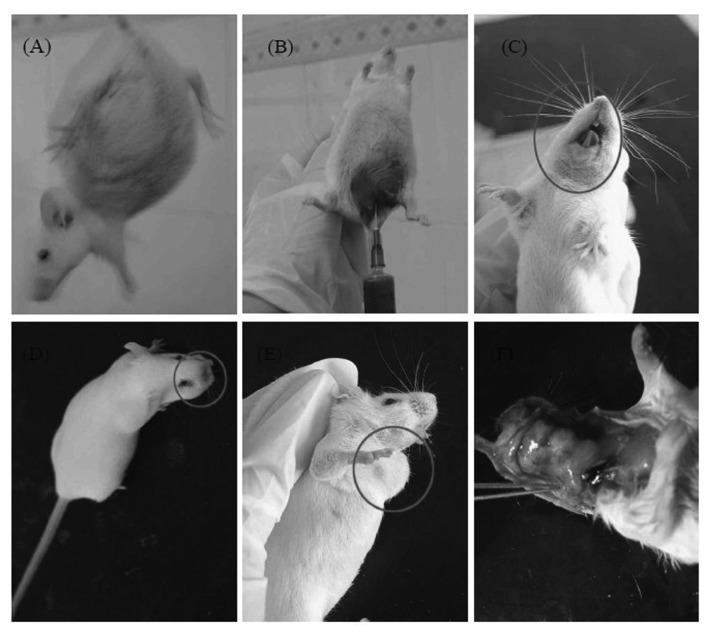
Changes in mice following U14 cell inoculation. (A) The mice were inoculated with U14 squamous cell carcinoma cells in the abdomen; (B) the carcinoma ascites were collected in the abdomen of mice; (C) ulceration in the angle of the mouth following inoculation with U14 cells; (D) primary foci formed following inoculation with U14 cells; (E) appearance of neck lymphangiectasis; (F) neck lymphangiectasis in anatomy.

U14 squamous cell carcinoma cells were obtained from the Chinese Academy of Medical Sciences (Beijing, China). The cancer cells were cultured in RPMI-1640 medium (Gibco Services Inc., Birmingham, MI, USA) supplemented with 10% fetal bovine serum (FBS) and 1% penicillin-streptomycin (Gibco Services Inc.) at 37°C in a humidified atmosphere containing 5% CO_2_ (model 311, Thermo Forma Inc., Waltham, MA, USA). The medium was changed 2–3 times each week. The *in vitro*-cultured U14 cells (5×10^6^ cells/mouse) were inoculated into the abdominal cavity of 7-week-old female ICR mice (Fig. 1A). After 1 week, the carcinoma ascites were collected and diluted in sterile saline at a concentration of 1×10^7^ cells/ml (Fig. 1B).

### Induction of buccal mucosa cancer

To establish the buccal mucosa cancer animal model, 50 mice were inoculated with 0.05 ml U14 cancer cell suspension (1×10^7^ cells/ml) on the buccal mucosa. Ten mice were bred as normal and acted as controls. Mice that succumbed to natural causes were collected and their buccal mucosa and lymph node tissues were determined. These experiments followed a protocol approved by the Animal Ethics Committee of Chongqing Medical University.

### Histological analysis of buccal mucosa cancer

Buccal mucosa and lymph node tissues were removed and embedded in paraffin for histological analysis with hematoxylin and eosin staining, as previously described (10).

### Reverse transcription-polymerase chain reaction (RT-PCR)

The mRNA expression of B cell lymphoma 2 (Bcl-2), Bcl-2-associated X protein (Bax), caspase-3, caspase-9, nuclear factor (NF)-κB, IκB-α, inducible nitric oxide synthase (iNOS), cyclooxygenase (COX)-2, matrix metalloproteinases (MMPs) and tissue inhibitors of MMPs (TIMPs) was assessed using RT-PCR. Total RNA was isolated using TRIzol reagent (Invitrogen Life Technologies, Carlsbad, CA, USA) according to the manufacturer’s instructions. The RNA was digested with RNase-free DNase (Roche, Basel, Switzerland) for 15 min at 37°C and purified using an RNeasy kit (Qiagen, Hilden, Germany) according to the manufacturer’s instructions. cDNA was synthesized from 2 *μ*g total RNA and incubated at 37°C for l h with AMV reverse transcriptase (GE Healthcare, Uppsala, Sweden) with random hexanucleotides, according to the manufacturer’s instructions. The primers used to specifically amplify the genes were as follows: forward, 5′-AAG CTG AGC GAG TGT CTC CGG CG-3′ and reverse, 5′-CAG ATG CCG GTT CAG GTA CTC AGT C-3′ for Bax; forward, 5′-CTC GTC GCT ACC GTC GTG ACT TGG-3′ and reverse, 5′-CAG ATG CCG GTT CAG GTA CTC AGT C-3′ for Bcl-2; forward, 5′-CAA ACT TTT TCA GAG GGG ATC G-3′ and reverse, 5′-GCA TAC TGT TTC AGC ATG GCA-3′ for caspase-3; forward, 5′-GGC CCT TCC TCG CTT CAT CTC-3′ and reverse, 5′-GGT CCT TGG GCC TTC CTG GTA T-3′ for caspase-9; forward, 5′-CAC TTA TGG ACA ACT ATG AGG TCT CTG G-3′ and reverse, 5′-CTG TCT TGT GGA CAA CGC AGT GGA ATT TTA GG-3′ for NF-κB; forward, 5′-GCT GAA GAA GGA GCG GCT ACT-3′ and reverse, 5′-TCG TAC TCC TCG TCT TTC ATG GA-3′ for IκB-α; forward, 5′-AGA GAG ATC GGG TTC ACA-3′ and reverse, 5′-CAC AGA ACT GAG GGT ACA-3′ for iNOS; forward, 5′-TTA AAA TGA GAT TGT CCG AA-3′ and reverse, 5′-AGA TCA CCT CTG CCT GAG TA-3′ for COX-2; forward, 5′-CTT CTT CAA GGA CCG GTT CA-3′ and reverse, 5′-GCT GGC TGA GTA CCA GTA-3′ for MMP-2; forward, 5′-TGG GCT ACG TGA CCT ATG AC-3′ and reverse, 5′-GCC CAG CCC ACC TCC ACT CC-3′ for MMP-9; forward, 5′-GTC AGT GAG AAG CAA GTC GA-3′ and reverse, 5′-ATG TTC TTC TCT GTG ACC CA-3′ for TIMP-1; forward, 5′-TGG GGA CAC CAG AAG TCA AC-3′ and reverse, 5′-TTT TCA GAG CCT TGG AGG AG-3′ for TIMP-2. The internal control gene, glyceraldehyde 3-phosphate dehydrogenase (GAPDH), was amplified with the following primers: forward, 5′-CGG AGT CAA CGG ATT TGG TC-3′ and reverse, 5′-AGC CTT CTC CAT GGT CGT GA-3′. Amplification was performed in a thermal cycler (Eppendorf, Hamburg, Germany) with cycles of denaturation. The amplified PCR products were run on 1.0% agarose gels and visualized by ethidium bromide (EtBr) staining (11).

### Western blot analysis

Total tissue protein was obtained with radioimmunoprecipitation assay (RIPA) buffer as described by Kim *et al* (12). Protein concentrations were determined using a Bio-Rad protein assay kit (Hercules, CA, USA). For western blot analysis, aliquots of the lysate containing 30–50 *μ*g protein were separated by sodium dodecyl sulfate-polyacrylamide gel electrophoresis (SDS-PAGE) and then electrotransferred onto a nitrocellulose membrane (Schleicher and Schuell Bioscience Inc., Keene, NH, USA). The membranes were subjected to immunoblot analysis and proteins were visualized by an enhanced chemiluminescence (ECL) method (GE Healthcare). The cell lysates were separated by 12% SDS-PAGE, transferred onto a polyvinylidene fluoride membrane (GE Healthcare), blocked with 5% skimmed milk and hybridized with primary antibodies (diluted 1:1,000). Antibodies against Bax, Bcl-2, caspase-3, caspase-9, NF-κB, IκB-α, iNOS, COX-2, MMPs and TIMPs were obtained from Santa Cruz Biotechnology Inc. (Santa Cruz, CA, USA), then incubated with the horse-radish peroxidase-conjugated secondary antibody (Santa Cruz Biotechnology Inc.) for 1 h at room temperature. Blots were washed three times with phosphate-buffered saline with Tween-20 (PBS-T) and then developed by ECL (Amersham Life Science, Arlington Heights, IL, USA).

## Results

### Changes following inoculation with U14 cells

Swelling was observed on the cheeks of ICR mice immediately after inoculation of U14 cells into the buccal mucosa of ICR mice (Fig. 1C). The swelling was observed on the inoculation area of the cheeks of ICR mice on days 1–5 and a similar inflammatory response was observed. On days 6–10, partial distention were observed on the inoculation area of the cheeks of ICR mice, with palpable masses; however, the lymph nodes on the necks were nonpalpable. In addition, primary foci gradually formed on the cheeks of ICR mice. Ulceration occurred on the cheek surface of certain mice and cervical lymph node swelling was identified in several ICR mice on days 11–15 (Fig. 1D). On days 16–20, the primary foci were further developed on the cheeks of ICR mice and cervical lymph node swelling was observed in certain mice (Fig. 1E). After 20 days, the inoculated cheek was larger than the cheek on the other side. Skin surface erosion occurred on certain mice and primary foci of the carcinoma were present. In addition, lymph node enlargement was observed in 88.8% of ICR mice to varying extents. Tumors were formed in the cheek of all ICR mice following inoculation, with no regression in all cases. The average survival time of ICR mice following inoculation was 16.40±4.45 days; the longest survival time was 25 days (Table I). Lymph node enlargement was observed on the necks of partial ICR mice 15 days after inoculation. Lymph node enlargement to varying extents was observed on all tumor-formed ICR mice on day 20 and the pathological results revealed that 8 ICR mice presented lymph node metastasis at a rate of 53% (Table II and Fig. 1F).

### Histological staining

**Figure 2:**
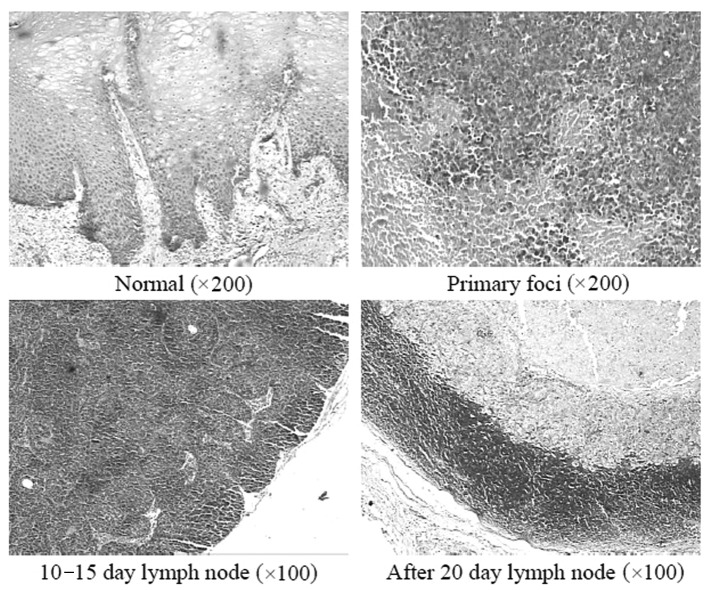
Histology of buccal mucosa and lymph node tissues induced by inoculation of U14 squamous cell carcinoma cells in mice.

H&E staining revealed that U14 cells firstly grew along the long axis of the muscle in the inter-muscular space, then gradually infiltrated into the muscles surrounding the intermusclar space and submucosa. Finally, the constant infiltration damaged almost all the muscle tissue and the majority of the submucosal tissue, and necrosis was observed in certain tumor areas (Fig. 2).

On days 10–15, there were lymphoid follicles in the lymph node demonstrating lymph node hyperplasia in the mice. After 15 days, neck lymph node metastasis was observed and after 20 days, the number of metastatic foci in the neck lymph nodes increased at the rate of 53%. The early tumor cells were in dense piles or aligned in the marginal sinus. In the later stage, they developed from the marginal sinus to the intermediate sinus and modularly sinus. The whole lymph node structure of the severe cases was damaged, with little lymphatic tissue left and necrosis in the central part of the carcinoma tissue. In addition, carcinoma cell emboli were observed in afferent and efferent lymphatic vessels in the lymph nodes of certain mice (Fig. 2).

### Bax, Bcl-2 and caspase expression

**Figure 3:**
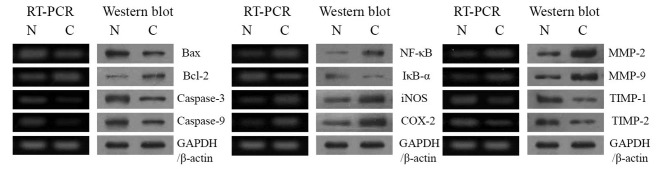
mRNA and protein expression of Bax, Bcl-2, caspases, NF-κB, IκB-α, iNOS, COX-2, MMPs and TIMPs in buccal tissues. N, normal group; C, cancer (inoculated with U14 squamous cell carcinoma cells) group. Bcl-2, B cell lymphoma 2; Bax, Bcl-2-associated X protein; NF, nuclear factor; iNOS, inducible nitric oxide synthase; COX cycooxygenase; MMP, matrix metalloproteinase; TIMP, tissue inhibitors of MMPs; GAPDH, glyceraldehyde 3-phosphate dehydrogenase.

To determine the mechanisms of buccal mucosa cancer, the expression of Bax, Bcl-2 and caspase genes in buccal mucosa tissues was determined by RT-PCR and western blot analyses. As shown in Fig. 3A, in the group treated with U14 cells (cancer-induced group), the Bax, Bcl-2, caspase-3 and caspase-9 genes demonstrated significant changes. We identified a decrease in Bax, an increase in Bcl-2 and a decrease in caspases in terms of mRNA and protein expressions, compared to the normal groups.

### NF-κB, IκB-α, iNOS and COX-2 expression

RT-PCR and western blot analyses were also conducted to investigate the level of inflammation resulting from gene regulation of inflammatory mediators, including NF-κB, IκB-α, iNOS and COX-2. As shown in Fig. 3B, NF-κB, iNOS and COX-2 inflammatory mediators were hardly detected in the normal group, while IκB-α was detected in the normal group. The mRNA and protein expression of NF-κB was increased, while the IκB-α mRNA level was decreased in the cancer-induced group. Additionally, mRNA and protein expression of COX-2 and iNOS gradually increased following inoculation of U14 cells.

### MMP and TIMP expression

RT-PCR and western blot analyses were conducted to determine whether metastasis in the buccal mucosa cancer model was a result of gene regulation of metastatic mediators, specifically MMPs (MMP-2 and MMP-9) and TIMPs (TIMP-1 and TIMP-2). As shown in Fig. 3C, significantly increased mRNA expression of MMP-2 and MMP-9 and decreased expression of TIMP-1 and TIMP-2 was observed in the cancer-induced group.

These changes in mRNA and protein expression demonstrate that inoculation of U14 cells effectively produces cancer, inflammation and metastasis *in vitro*.

## Discussion

U14 cells are a squamous cell carcinoma cell line (6) that was ectopically induced by treating the uterine cervix with methylcholanthrene thread. They are able to induce cancer by implantation into the subskin of adult mice (13). In the early stages, its structure is similar to a carcinosarcoma. It is considered an undifferentiated carcinoma, with a metastasis rate of 95% in the lymph node and 80% in the lungs. Moreover, it is a bidirectional metastasis tumor strain and an optimum model that is widely used in studies on tumor metastasis, invasion, recurrence and drug screening. This tumor strain is characterized by low cell differentiation, high proliferation, extremely strong infiltration and metastasis. Additionally, its animal inoculation survival rate is capable of reaching 100% (6).

With the growth of tumor tissue, the number of open and expanding peri-cancer lymphatic vessel increases. The vessel wall has an incomplete structure with an evident gap or clearance. This is usually observed in the area where tumor cells are concentrated as it allows the tumor cells to enter into the lymphatic lumen. This occurs in two ways: a single carcinoma cell or clusters of carcinoma cells squeeze into the lymphatic lumen through the open clearance of the complete vessel wall or carcinoma cells enter into lymphatic lumen by damaging a part of or the majority of the vessel wall (14). The former is usually observed 15 days after tumor inoculation and the latter, which is the main way that carcinoma cells enter the lymphatic lumen, is observed 20 days after inoculation. In the meantime, more metastasis foci occur on neck lymph nodes.

Apoptosis is a fundamental cellular event and understanding its mechanisms of action is likely to enable this process to be used in tumor diagnosis and therapy (15). In a healthy cell, the anti-apoptotic protein Bcl-2 is expressed on the outer mitochondrial membrane surface (16). Caspases form a proteolytic network within the cell, whereby upstream initiator caspases are activated early in the apoptotic process (caspase-9) and in turn activate other downstream caspases (caspase-3). Cytochrome *c* and procaspase-9 processing is highly dependent on caspase-3, placing this caspase in a central position as a regulator of essential apoptotic pathways in cancer cells (17). Caspase-3 also plays a role as an amplifier of apoptotic signals, by cleaving Bcl-2 (18). COX-2 plays an important role in colon carcinogenesis, and NOS, along with iNOS may be a good target for the chemoprevention of cancer (19). NF-κB is one of the most ubiquitous transcription factors and regulates the expression of genes required for cellular proliferation, inflammatory responses and cell adhesion (20). NF-κB is present in the cytosol where it is bound to the inhibitory protein, IκB.

Metastasis is defined as the spread of cancer cells from one organ or area to another adjacent organ or location (21). MMPs, a family of zinc-dependent endopeptidases, play an important role in tumorigenesis and metastasis. MMPs are able to cleave virtually all extracellular matrix (ECM) substrates. Degradation of the ECM is a key event in tumor progression, invasion and metastasis (22). Among the MMP family members, MMP-2 and -9 are important for cancer invasion (23). In fact, inhibition of MMP activity is useful for controlling tumorigenesis and metastasis (24). TIMPs are naturally occurring inhibitors of MMPs, which prevent catalytic activity by binding to activated MMPs, thereby blocking ECM breakdown (25). Disturbances in the ratio between MMPs and TIMPs have been observed during tumorigenesis (26). MMP-2 and -9 are key factors in cancer cell invasion and metastasis *in vitro* (27). Spontaneous and experimental metastasis to the liver is decreased in mice overexpressing TIMP1 and increased in mice expressing antisense TIMP-1 mRNA (28). Ectopic overexpression of TIMP-1 in the brains of transgenic mice also reduces experimental metastasis to the brain (29). In the present study, the apoptotic, inflammatory and metastatic gene expression results demonstrated that inoculation of U14 cells induces buccal mucosa cancer *in vivo*.

In this study we identified that carcinoma metastasis firstly encroaches on the marginal sinus, then infiltrates to the cortex and medulla. The infiltration continues until normal lymph node structure is completely damaged. Our results are in agreement with previous studies. Furthermore, experimental results revealed that, with the reactive hyperplasia of the lymph node, degeneration and necrosis of large areas is observed in the tumor area. The neck lymph node of the cancer-induced mice increased as the experimental time increased and it became significantly larger after day 15. However, microscopic inspection revealed that not all the lymph nodes were affected by metastasis or proliferation of tumor cells and more than half of them were affected by the reactive hyperplasia of lymph nodes. This indicates that lymph nodes experience immune rejection of the tumor following tumor antigen stimulation (30). However, further study is required to determine the biological barrier impact of the lymph node on tumor metastasis.

In this study, carcinoma neck lymph node metastasis animal models were established. After 20 days, the cheek tumor formation rate reached 100% and the neck lymph node metastasis rate was 53%. The metastasis of the tumor to the lymph node occurs as follows: the carcinoma encroaches on the marginal sinus; then infiltrates the cortex and medulla. The infiltration continues until the normal lymph node structure is completely damaged. This animal model may be used in medical research on buccal mucosa cancer and cervical lymph node metastasis.

## Figures and Tables

**Table I:** Overall observations of mice inoculated with U14 squamous cell carcinoma cells.

Time (days)	No. mice that survived	Formation of enclosed buccal mass	Lymphangiectasis
1–5	48	12	0
6–10	42	38	0
11–15	29	26	2
16–20	15	15	9
>20	9	9	8

n=50.

**Table II:** Tumor formation rate and lymph node metastasis rate in ICR mice inoculated with U14 squamous cell carcinoma cells.

Time (days)	Tumor formation rate^a^ (%)	Lymph node metastasis rate^b^ (%)
11–15	79.3 (23/29)	0
16–20	100 (15/15)	53.3 (8/15)

a tumor formation/mice that survived;

b lymph node metastasis/tumor formation.
